# Degradation-structure-mechanical relationships of PLGA suture anchors and PCL coating-mediated mechanical preservation via degradation-induced crystallization

**DOI:** 10.3389/fbioe.2026.2003985

**Published:** 2026-10-29

**Authors:** Yao Lu, Jingbo Xu, Nan-Nan Li, Bing Hu, Songbiao Xue, Qingxia Song

**Affiliations:** 1 College of Medical Devices, Zhejiang Pharmaceutical University, Ningbo, China; 2 Ningbo Key Laboratory of Intelligent Implantable and Interventional Medical Devices, Zhejiang Pharmaceutical University, Ningbo, Zhejiang, China; 3 Ningbo Weierkaidi Medical Devices Co., Ltd., Ningbo, China

**Keywords:** degradation kinetics, degradation-induced crystallization, *in vivo* biocompatibility, PCL coating, PLGA suture anchor, structure-mechanical correlation

## Abstract

PLGA suture anchors are widely used for orthopedic soft-tissue fixation, yet their degradation-structure-mechanical evolution and the potential for coating-mediated mechanical regulation remain incompletely characterized. Here, we investigated the *in vitro* degradation of uncoated PLGA anchors over 24 weeks, structure-mechanical correlations through molecular weight-compression force coupling, and whether PCL coatings (5%, 10%, 20%) preserve mechanical performance through degradation-induced crystallization, complemented by a preliminary rat fascia-defect *in vivo* assessment. Uncoated anchors showed staged degradation: mass loss was modest through 8 weeks (<5.4%) but accelerated after 16 weeks (>35%), while Mn declined from ∼185,000 to ∼105,000 Da by 12 weeks and further thereafter. Compression Fmax remained at or above its initial value through 16 weeks despite ongoing chain scission, indicating structural-mechanical decoupling in which molecular degradation preceded macroscopic mechanical change. DSC confirmed that the material remained amorphous, with Tg remaining approximately 65 °C during degradation, and SEM revealed progressive surface pore formation. Among coated groups, PCL10% preserved mechanical performance best, with an initial ΔHm of about 46 J/g (Xc ≈ 33%) that increased further after 4 weeks, indicating a crystalline physical crosslink network; PCL5% degraded too rapidly and PCL20% showed no corresponding advantage. At Day 8 *in vivo*, blood and biochemical parameters showed individual deviations (n = 2 per group), and the implant caused notable local tissue reactions. Histology showed HE inflammatory cell density lower in sham (∼340–375 cells/mm^2^) than suture (∼743–813) and anchor (∼775–882), and CD206-positive densities remained below 10 cells/mm^2^ in all groups, with no inferential statistics. These findings define the structure-mechanical relationship governing PLGA anchor degradation and show that PCL10% coating preserves compression performance, providing a design rationale for absorbable anchor coatings, with boundaries set by the small *in vivo* sample, descriptive histology, batch differences, and single-point GPC data.

## 1 Introduction

Suture anchors are widely used for soft-tissue-to-bone fixation in orthopedic surgery, and their design and material choices directly affect fixation reliability (Barber et al., 2006). Absorbable anchors avoid the need for hardware removal and reduce long-term implant-associated complications; among biodegradable polymers, poly (lactic-co-glycolic acid) (PLGA) is attractive because its degradation profile and mechanical properties can be tailored through composition and molecular weight (Surya et al., 2021; Wang et al., 2023). PLGA-based implants and *in situ* forming depots have also been widely investigated in biomedical applications (Wang et al., 2025). However, absorbable implants must retain sufficient fixation strength during tissue healing while resorbing predictably thereafter (Wang et al., 2023).

The degradation of PLGA proceeds mainly by bulk hydrolysis, in which ester bonds are cleaved throughout the hydrated matrix; molecular weight therefore declines before substantial mass loss, and macroscopic mechanical deterioration generally lags behind molecular chain scission (Surya et al., 2021; Palkar and Kuksenok, 2022; Ran et al., 2024). Amorphous polyesters undergo bulk hydrolytic chain scission, whereas semicrystalline polyesters hydrolyze preferentially in amorphous regions (Palkar and Kuksenok, 2022; Ran et al., 2024). For load-bearing devices such as anchors, this temporal mismatch between molecular degradation and mechanical performance is a key design challenge, and its full trajectory in a suture-anchor geometry remains incompletely characterized (Barber et al., 2006; Wang et al., 2023; Whitfield et al., 2025).

Coating strategies offer a route to modulate degradation-mechanical coupling without redesigning the anchor. Poly (ε-caprolactone) (PCL) is a semicrystalline polyester widely used in biomedical materials and fibers (Meng, 2025; Xue et al., 2025). Surface coating has been shown to prolong degradation and maintain the mechanical strength of surgical sutures *in vivo*, indicating that coating-mediated mechanical preservation is feasible (Wu et al., 2022). Yet the optimal PCL coating composition and its crystallization-dependent mechanical benefit for PLGA anchors have not been systematically established.

In this study, we investigated the degradation-structure-mechanical evolution of uncoated PLGA suture anchors over 24 weeks of *in vitro* hydrolysis (Figures 1–3) and evaluated whether PCL coatings (5%, 10%, and 20%) could preserve compression performance through degradation-induced crystallization (Figure 4). We further performed a preliminary *in vivo* assessment in a rat fascia defect model to examine acute-phase systemic parameters and local tissue response at Day 8 (Figures 5–7). Our results show that molecular chain scission precedes macroscopic mechanical change, that PCL10% coating most effectively preserves compression performance through crystallization-mediated compensation, and that the *in vivo* data provide preliminary safety evidence with descriptive histological cell-density analysis. The small sample size and batch differences define clear boundaries for these conclusions.

**FIGURE 1:**
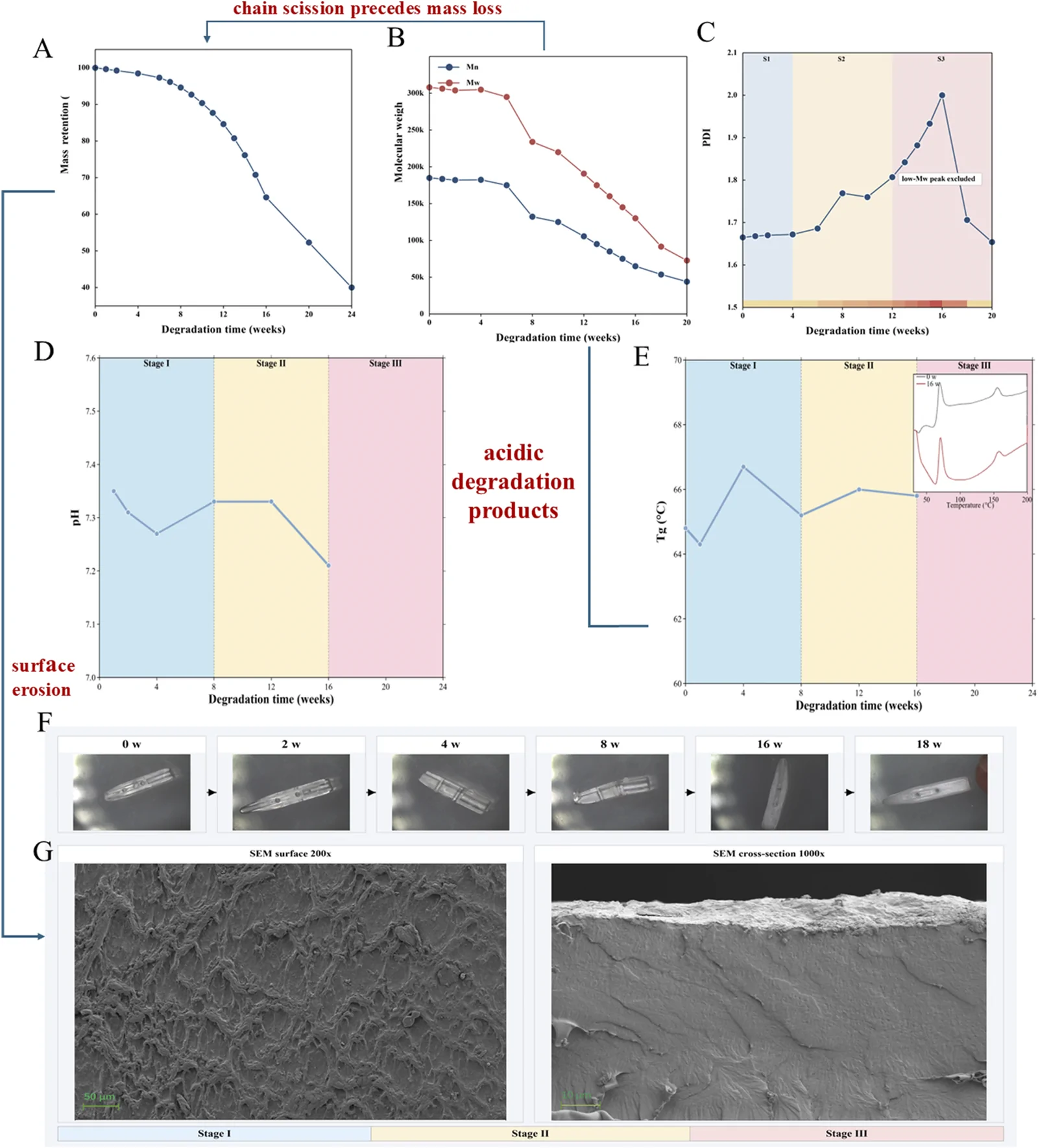
Degradation kinetics of uncoated PLGA suture anchors in PBS (pH 7.4, 37 °C, 60 rpm). **(A)** Mass retention over 0–24 weeks. **(B)** Mn and Mw as a function of degradation time. **(C)** Polydispersity index (PDI). **(D)** pH of the degradation medium at each sampling point. **(E)** DSC-derived Tg. **(F)** Stereomicroscopic surface morphology at 0, 2, 4, 8, 16, and 18 weeks. **(G)** Representative SEM images: g1, surface at 200×; g2, cross-section at 1,000×.

**FIGURE 2:**
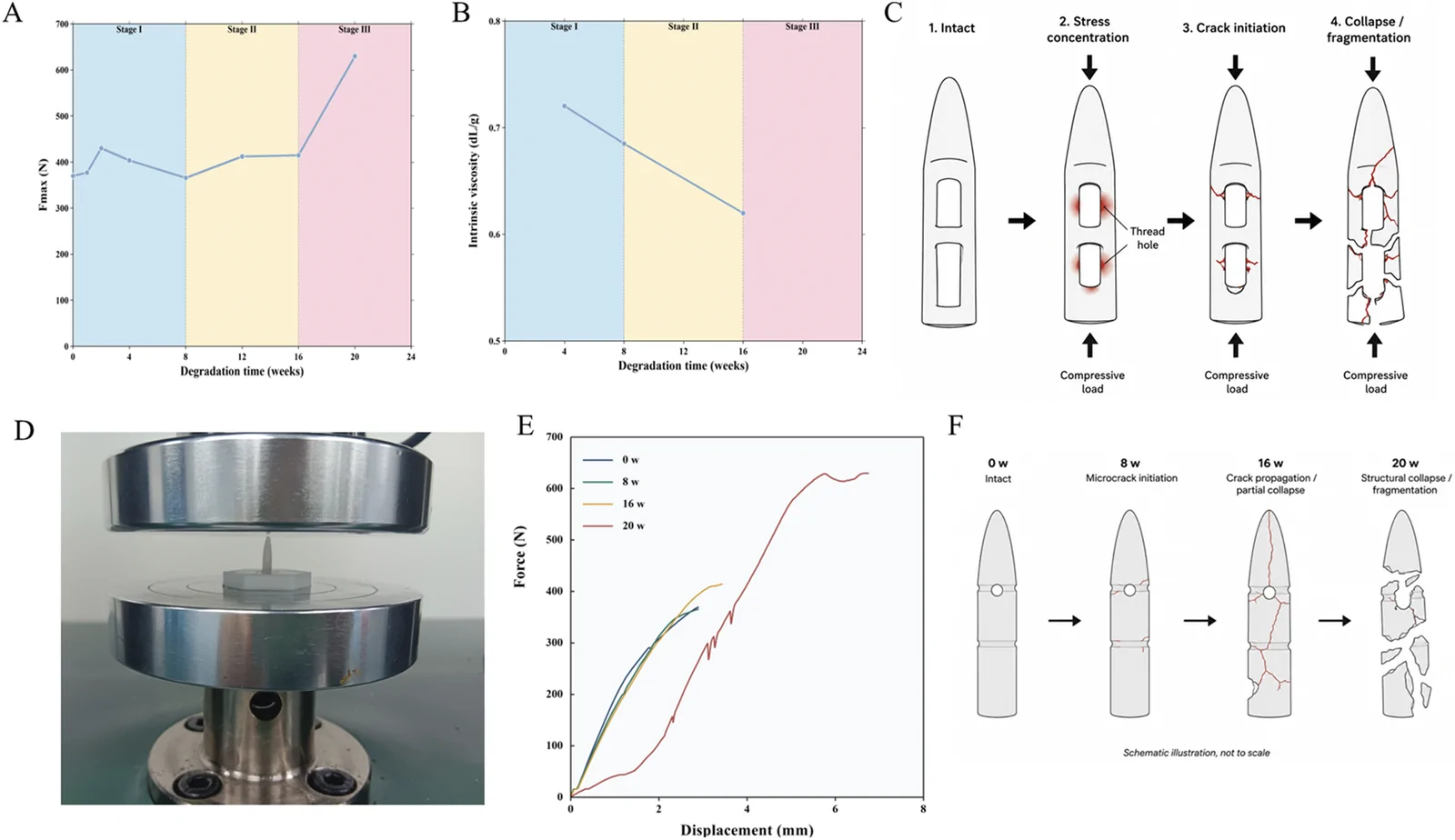
Mechanical evolution of uncoated PLGA suture anchors during *in vitro* degradation. **(A)** Compression Fmax and retention ratio versus degradation time (0–20 weeks). **(B)** Intrinsic viscosity as a function of degradation time. **(C)** Compression failure mechanism of uncoated PLGA anchors. **(D)** Photographs of the compression testing setup. **(E)** Representative load–displacement curves at 0, 8, 16, and 20 weeks. **(F)** Compressive failure evolution of uncoated PLGA suture anchors during degradation.

**FIGURE 3:**
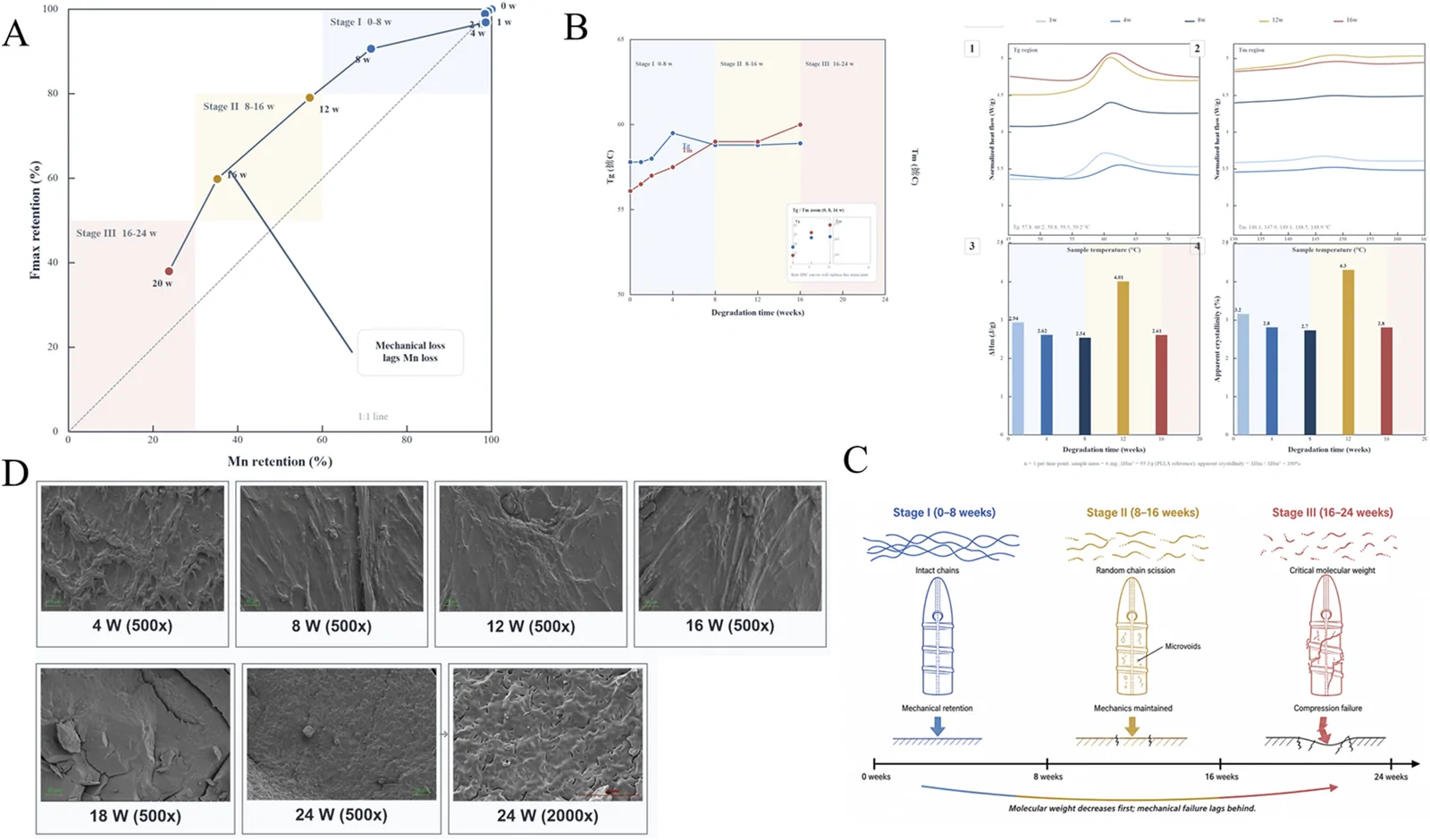
Structure–mechanical correlations of uncoated PLGA suture anchors during degradation. **(A)** Mn retention versus compression Fmax retention, with degradation time labeled on each data point; dashed line indicates 1:1 reference. **(B)** Tg as a function of degradation time. **(C)** Schematic illustration of chain scission progressing from intact chains (Stage I, 0–8 weeks) through random chain scission (Stage II, 8–16 weeks) to critical molecular weight (Stage III, 16–24 weeks), with delayed mechanical failure. **(D)** Representative SEM surface images at 4, 8, 12, 16, and 24 weeks (500×).

**FIGURE 4:**
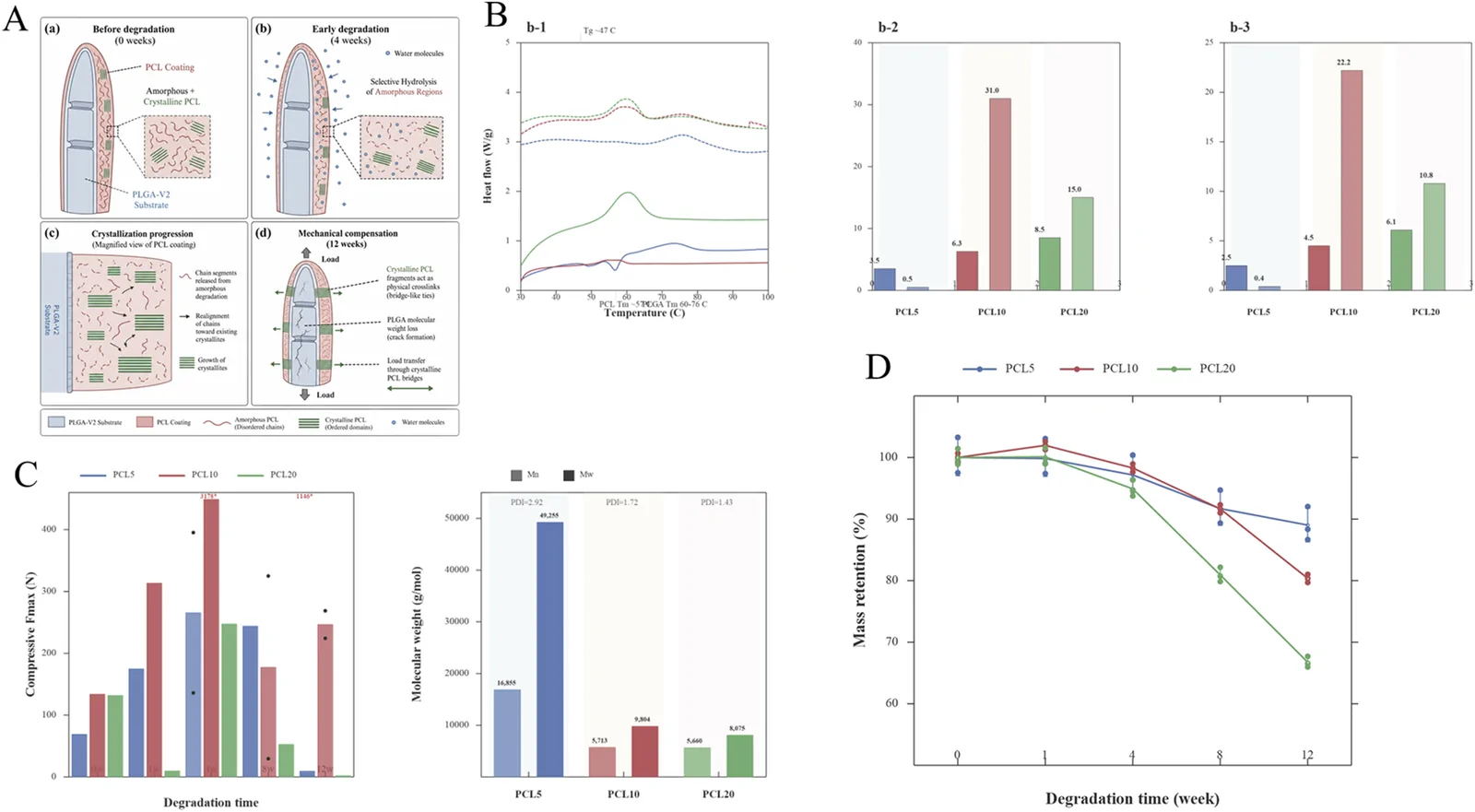
PCL10% coating preserves mechanical performance through degradation-induced crystallization. **(A)** Mechanism schematic: amorphous PCL hydrolysis preferentially degrades non-crystalline regions, while retained crystalline domains form a physical crosslink network that compensates for molecular weight loss. **(B)** DSC results of PCL5%, PCL10%, and PCL20% coatings showing ΔHm and crystallinity after 4 weeks of degradation. **(C)** Compression Fmax retention (top) and GPC molecular weight data at 12 weeks (bottom). Open symbols denote suspected outliers. **(D)** Mass retention of PCL-coated groups during degradation up to 12 weeks.

**FIGURE 5:**
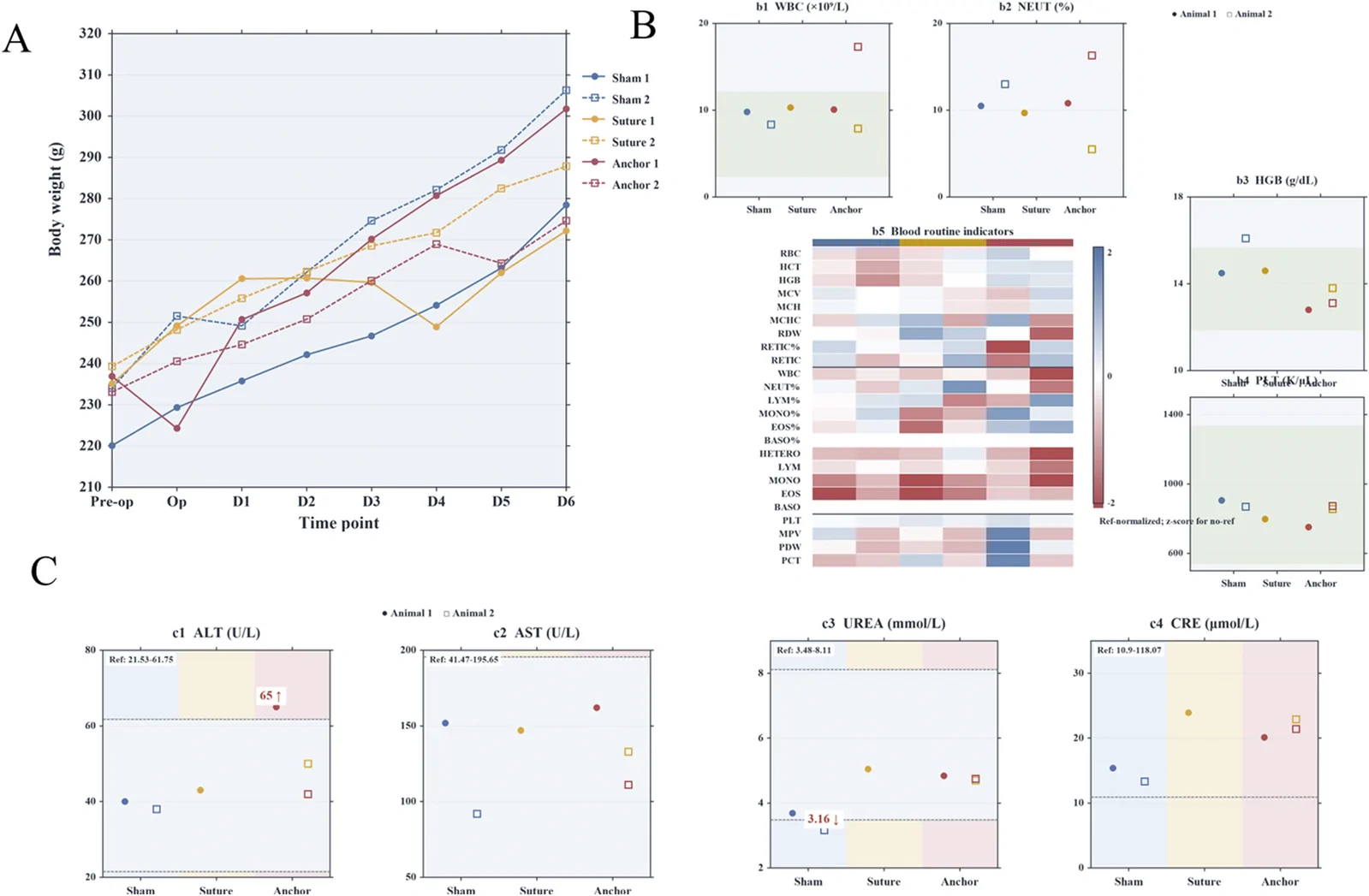
Acute-phase systemic parameters in SD rats at Day 8 post-surgery. **(A)** Body weight changes during the observation period. **(B)** Key hematological parameters. **(C)** Serum biochemical parameters (ALT, AST, UREA, and CRE). All data are presented as individual data points (n = 2 per group); no between-group statistical analysis was performed.

**FIGURE 6:**
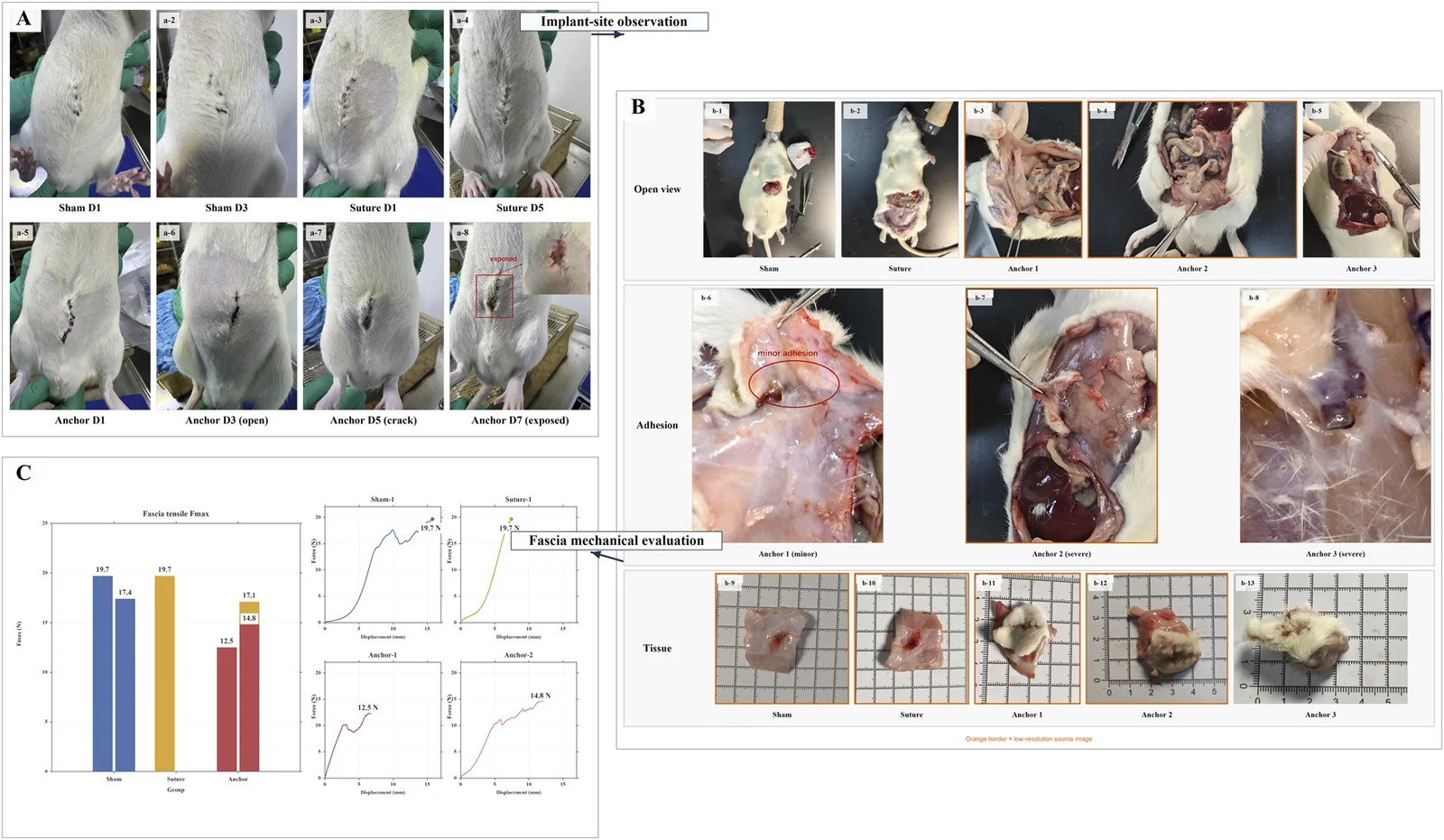
*In vivo* gross observation and fascia mechanical evaluation at Day 8 post-surgery. **(A)** Representative wound healing progression from Day 1 to Day 8. **(B)** Gross observation of subcutaneous tissue, fascia, and abdominal adhesion. **(C)** Implant-region force-displacement results for individual animals.

**FIGURE 7:**
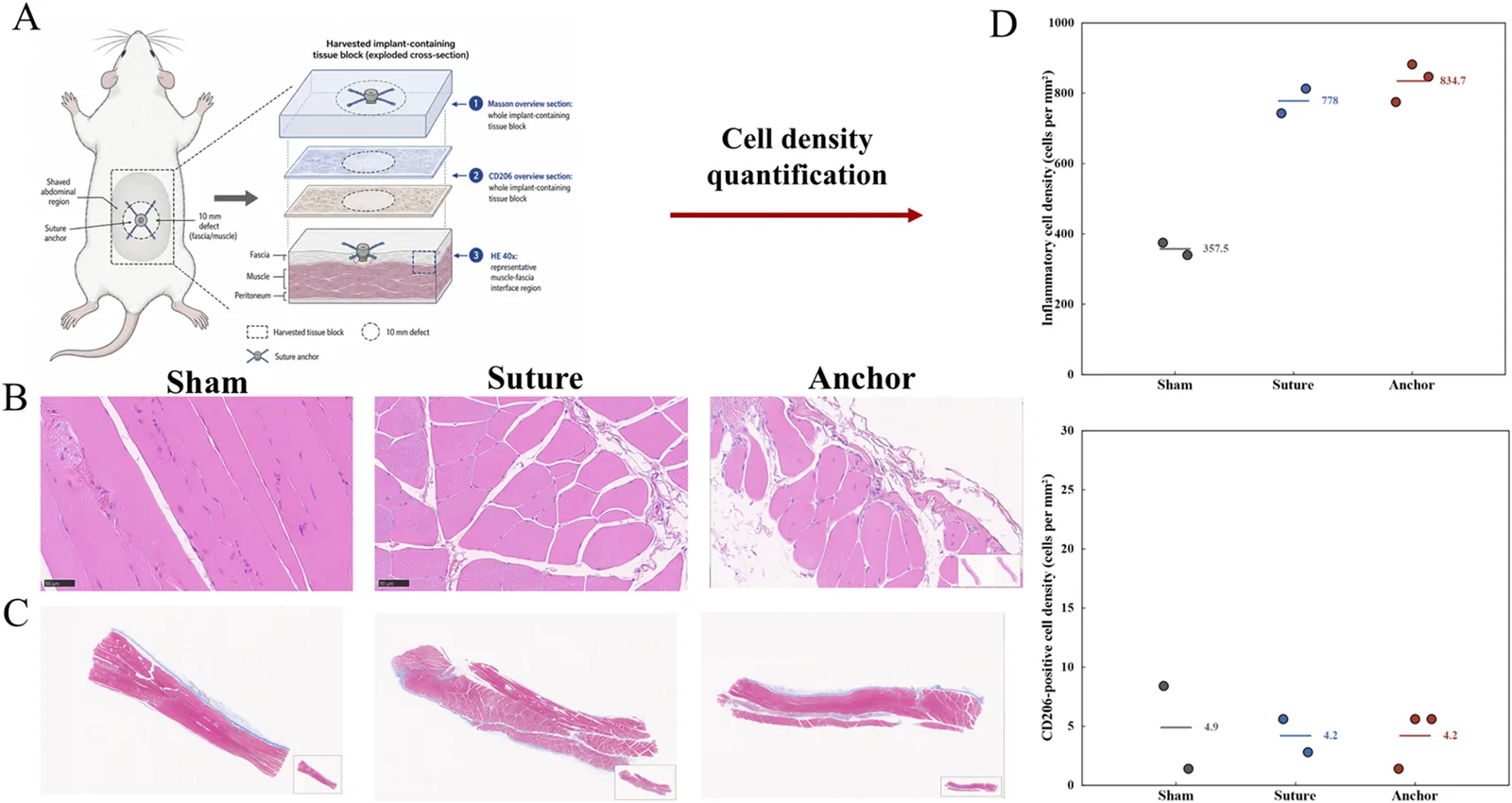
Histological evaluation of the implant region. **(A)** Schematic illustration of the implant site for anatomical orientation. **(B)** Representative HE-stained sections (40×) from the sham, suture, and anchor groups. **(C)** Representative Masson-stained sections showing collagen distribution. **(D)** HE-derived inflammatory cell density and CD206-positive cell density shown as individual animal data points (three non-overlapping fields per animal at 40×; FN = 22; field area = 0.2376 mm^2^). Morphological findings are qualitative, and cell densities are descriptive quantitative data; no between-group inferential statistics were performed. Scale bars and magnification are indicated on each image.

## 2 Methods

### 2.1 Materials and anchor preparation

Suture anchors were fabricated from a commercially purchased PLGA copolymer with an LA/GA ratio of 75:25 (the copolymer was not synthesized or blended in-house). The initial molecular weight of the PLGA was Mn ≈ 185,000, Mw ≈ 308,000, and PDI = 1.665. The anchors were produced by injection molding; the anchor body measured φ3.26 mm × 16.88 mm, and its surface was mechanically polished. All anchors were produced from the same PLGA formulation using an identical manufacturing process, with controlled dimensions. For animal experiments, anchors were trimmed to 1.2 cm, four anchors were used per defect, the anchor spacing was 5 mm, and 3–0 PGA sutures were pre-threaded. Before use, anchors were ultrasonically cleaned in absolute ethanol for 10 min, rinsed three times with deionized water, and vacuum-dried at 40 °C for 2 h.

### 2.2 *In vitro* degradation experiment

Uncoated PLGA anchors were immersed in phosphate-buffered saline (PBS, pH 7.4) for *in vitro* degradation. PBS was prepared according to ASTM F1635-16: NaCl 8.0 g, KCl 0.2 g, Na_2_HPO_4_·12H_2_O 2.9 g, KH_2_PO_4_ 0.2 g, and deionized water to a final volume of 1 L. Samples were placed in centrifuge tubes containing 50 mL PBS and incubated with shaking at 37 °C ± 0.5 °C and 60 rpm. The PBS was replaced weekly, and the pH of the medium was measured and recorded before each replacement. The overall degradation study spanned 0–24 weeks, but individual assays were measured at the time points indicated in each figure.

Sampling time points were set according to the specific characterization parameter and covered the entire degradation process. For the compression mechanical time series, the original test data were used with sampling at 0, 1, 2, 4, 8, 12, 16, and 20 weeks; the values in the text are fully consistent with Figure 2 and were taken from the original mechanical test dataset. Degraded samples were used for measurements of mass change, molecular weight, intrinsic viscosity, pH, thermal properties, surface morphology, and compression mechanics.

### 2.3 Characterization methods

Mass retention was calculated from the dry-weight change of samples. After sampling, samples were gently rinsed with deionized water and vacuum-dried to constant weight before weighing. Mass loss was calculated as (W_0_ − W_a_)/W_0_ × 100% according to GB/T 16,886.13–2017.

Molecular weight and molecular weight distribution were determined by GPC using an Agilent 1260II gel permeation chromatograph with an organic-phase GPC column. Tetrahydrofuran (THF, chromatographic grade) was used as the mobile phase at 1.0 mL/min, the column temperature was 35 °C, and the injection volume was 20 μL. Samples were dissolved in THF to approximately 2 mg/mL, filtered, and injected. Calibration was performed with narrow-distribution polystyrene standards, and Mn, Mw, and PDI were recorded, with initial values of Mn ≈ 185,000, Mw ≈ 308,000, and PDI = 1.665. The procedure followed molecular exclusion chromatography (Chinese Pharmacopoeia 2025; Volume IV, General Chapter 0514) and YY/T 1806.1–2021.

Intrinsic viscosity was measured with an Ubbelohde viscometer. The pH of the degradation medium was recorded with a pH meter (PHB-4, Shanghai INESA).

DSC was performed with a PerkinElmer DSC4000 differential scanning calorimeter under nitrogen (20 mL/min). Dry samples (8.0 ± 1.0 mg) were sealed in aluminum crucibles and heated from 30 °C to 200 °C at 20 °C/min, followed by a 5-min isothermal hold at 200 °C. The glass transition temperature (Tg) was determined from this first heating scan. The procedure followed Chinese Pharmacopoeia 2025, Volume IV, General Chapter 0661, and YY/T 1806.1–2021.

Surface morphology was observed by stereomicroscopy (YYT-880E) and SEM. SEM recorded surface and cross-sectional morphology, and magnifications were labeled.

Compression mechanical properties were measured with a universal testing machine (UTM4304X, Shenzhen SANS) in axial compression mode at a loading rate of 2 mm/min and room temperature. Load-displacement curves and the maximum compressive load Fmax were recorded. Fmax retention was calculated as the ratio of the Fmax at each week to the Fmax at week 0. The compression data were derived from the original mechanical test dataset, and the values in the text are consistent with Figure 2. ISO 604 was used only as a framework for the loading rate; the procedure also followed ASTM F1635 and YY/T 1806.1–2021.

### 2.4 Preparation and characterization of PCL-coated anchors

PCL single-coated anchors were prepared on PLGA anchors by dip coating. PCL pellets (Mn = 80,000) were added to dichloromethane (DCM) and magnetically stirred (300 rpm, 25 °C, 6 h) until fully dissolved to prepare PCL solutions at concentrations of 5 wt%, 10 wt%, and 20 wt%. Anchors were immersed in the PCL solution for 60 s and withdrawn at 0.5 mm/s, allowed to evaporate in a fume hood for 15 min, vacuum-dried at 40 °C for 2 h to remove DCM, and then vacuum-dried at 40 °C for 24 h for curing. Only single PCL coatings were prepared in this study; HA and CS components were not used.

The initial thickness of the coated anchors was determined gravimetrically, calculated as (m_coated − m_original)/(ρ_PCL × A), where ρ_PCL = 1.15 g/cm^3^ and A is the anchor surface area. The initial thicknesses were 0.17, 0.22, and 0.34 mm for PCL5%, PCL10%, and PCL20%, respectively. The initial compressive strengths were 6.88, 11.93, and 11.38 MPa, and the initial elastic moduli were 71.37, 64.24, and 133.70 MPa.

PCL-coated anchors were tested under the same *in vitro* degradation conditions as uncoated anchors (PBS, pH 7.4, 37 °C ± 0.5 °C, 60 rpm), with sampling at 0, 1, 4, 8, and 12 weeks. Mass change, pH, DSC, GPC, compression Fmax, and elastic modulus were measured. The apparent crystallinity of the PCL coating was calculated as Xc = ΔHm/139.5% × 100%, where ΔHm is normalized to the PCL coating mass and 139.5 J/g is the melting enthalpy of fully crystalline PCL. All original compression Fmax values were retained; suspected outliers (PCL5% at 12 weeks and PCL10% at 4 weeks) were kept in the dataset and marked with open symbols in the figures rather than deleted.

### 2.5 Animal experiments

Animal experiments were approved by the Ethics Committee of Zhejiang Pharmaceutical University (approval No. ZYLL 202605005).

Male Sprague-Dawley (SD) rats (8 weeks old, 250–300 g, SPF grade) were housed in a standard barrier environment. The trimmed anchors were fixed onto the fascia surface during surgery; at sampling, some anchors were found to have partially migrated into the underlying muscle layer because of animal movement. A 10 mm circular fascia defect was created adjacent to the linea alba, and rats were randomly divided into three groups: sham group (n = 2, only a 10 mm fascia defect without anchor fixation), suture group (n = 2, fascia defect closed with 3–0 PGA sutures), and PLGA anchor group (n = 3, four PLGA anchors trimmed to 1.2 cm implanted per defect, with 5 mm spacing and pre-threaded 3–0 PGA sutures). Systemic safety analyses (body weight, hematology, and serum biochemistry) were performed in n = 2 animals per group, whereas histological and adhesion evaluations included all three anchor-group animals. At Day 8 after surgery, rats were deeply anesthetized by isoflurane inhalation (2%–3% for induction and 1.5%–2% for maintenance) and euthanized by terminal blood collection via cardiac puncture under deep anesthesia; samples were then collected.

After surgery, wound healing, abdominal adhesion score (0–3), body weight change, and licking/biting behavior were observed and recorded daily. At sampling, whole blood was collected and complete blood count parameters (WBC, RBC, HGB, HCT, MCV, MCH, MCHC, PLT, and differential leukocyte counts) were measured with an automated hematology analyzer. Serum was separated and serum biochemical parameters (ALT, AST, UREA, CRE) were measured with an automated biochemistry analyzer. Fascia specimens were subjected to uniaxial tensile testing on a universal testing machine at 10 mm/min, and the maximum force and force-displacement curves were recorded. All animal procedures followed the guidelines for the care and use of laboratory animals.

### 2.6 Histological evaluation

Fascia and implant-region specimens were fixed in 4% paraformaldehyde, embedded in paraffin, and sectioned at 5 μm thickness. Sections were stained with HE, Masson, and CD206 immunohistochemistry, and observed under light microscopy to assess skeletal muscle morphology, collagen distribution, and CD206-positive signals in the implant region. For HE staining, inflammatory cells within each field were counted; for CD206 immunohistochemistry, DAB-positive cells were counted. Three non-overlapping fields per animal were selected under a ×40 objective (FN = 22), and cell density (cells/mm^2^) was calculated from the field area. Values were first averaged per animal and then presented as individual animal values and ranges. All histological images included scale bars and magnifications. Morphological findings were described qualitatively, and cell densities were presented as descriptive quantitative data without inflammation scoring, fibrosis scoring, or between-group statistical testing.

### 2.7 Statistical analysis

Data were analyzed with SPSS 26.0 or Origin. For all *in vivo* data, individual data points and descriptive statistics were used, and no inferential statistical tests were performed. Suspected outliers were retained with their original values and noted in the figure legends.

## 3 Results

The overall study design, encompassing anchor preparation, *in vitro* degradation scheme, PCL coating strategy, and *in vivo* evaluation, is illustrated in Figure 8.

**FIGURE 8:**
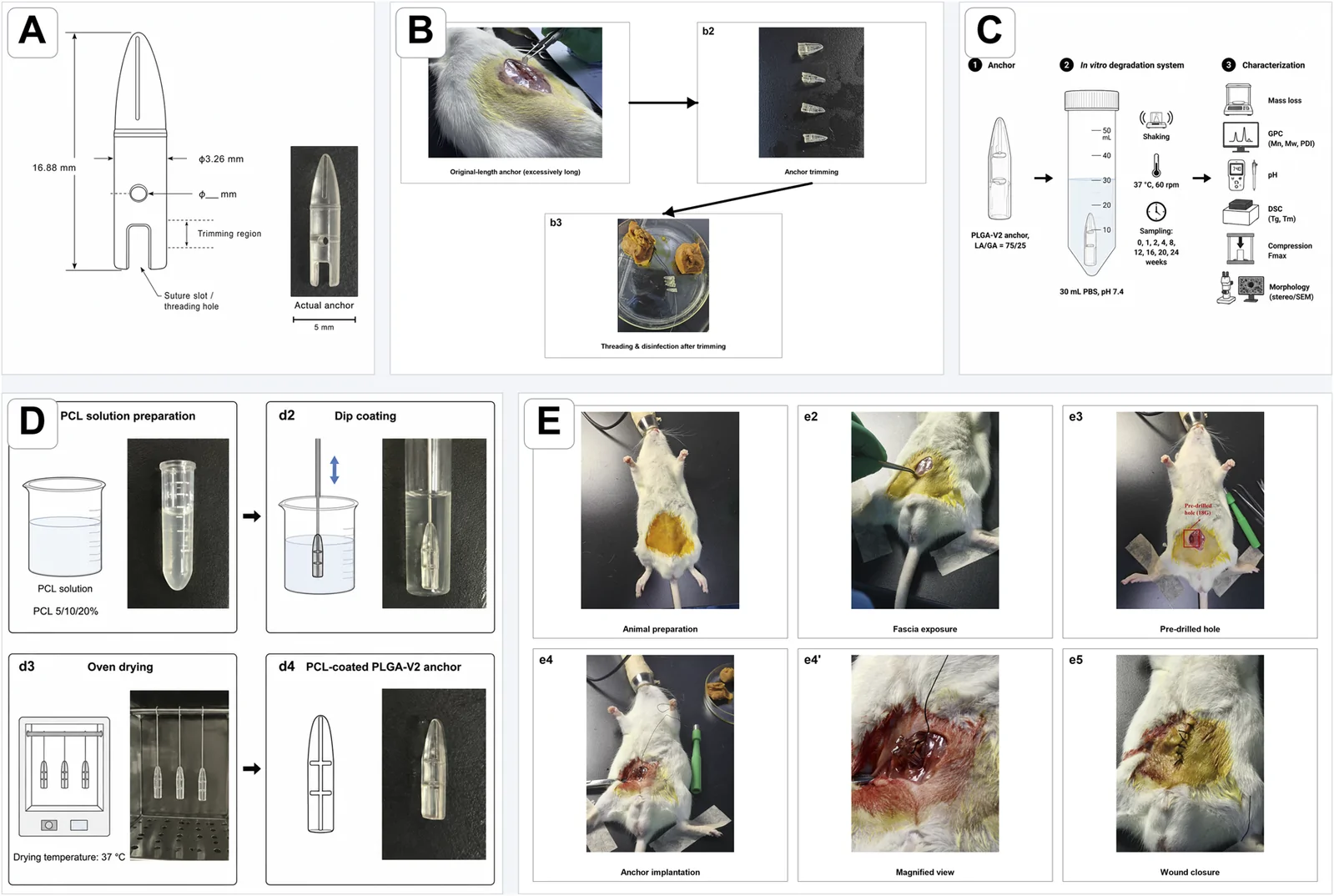
Schematic overview of the PLGA suture anchor system. **(A)** Anchor geometry and key structural features. **(B)** Anchor preparation workflow: b1, as received with excess length; b2, trimmed anchor; b3, threaded and disinfected anchor. **(C)** *In vitro* degradation scheme (PBS, 37 °C, 60 rpm, 0–24 weeks). **(D)** PCL coating strategy with three concentration levels (5%, 10%, 20%). **(E)** Rat abdominal fascia defect model: e1, skin preparation and hair removal; e2, fascia exposure and 10 mm circular defect creation; e3, predrilling; e4, anchor implantation (4 anchors per defect, 5 mm spacing); e5, wound closure.

### 3.1 *In vitro* degradation kinetics of uncoated PLGA anchors

Uncoated PLGA anchors showed time-dependent degradation in PBS (pH 7.4, 37 °C, 60 rpm) (Figure 1). The initial anchor mass was 0.130 g. Mass loss was slow during the first 8 weeks (5.38% loss at week 8), accelerated after week 8, reached 35.4% at week 16, and substantial weight loss continued at weeks 20 and 24. Mn declined progressively from an initial 185,000 Da to 105,528 Da at week 12 (57.0% retention), decreased further after week 16 and continued to fall at later time points. PDI first increased and then decreased from an initial 1.665, showing a broadening-then-narrowing trend that indicates concurrent chain scission and dissolution of low-molecular-weight species. The pH of the degradation medium remained near neutral throughout the 16 weeks (approximately 7.2–7.4), consistent with the buffering capacity of PBS and the weekly medium replacement. DSC showed that Tg remained approximately 65 °C from week 0 to week 16, and the material remained amorphous throughout degradation.

Stereomicroscopic morphology (Figure 1F) showed a smooth surface at week 0, local microcracks at week 4, crack propagation after week 8, and macroscopic pores and surface erosion at weeks 16–18. SEM (Figure 1G) further confirmed the evolution from an initially dense and smooth surface to a porous, cracked structure, and cross-sections showed degradation advancing layer by layer from the outside inward.

### 3.2 Mechanical evolution of uncoated PLGA anchors

Compression testing covered 0–20 weeks (Figure 2). The maximum compressive load (Fmax) was 369.96 N at week 0 and fluctuated rather than decreased monotonically during weeks 1–16: the values at weeks 1 (377.0 N), 4 (403.6 N), 12 (411.9 N), and 16 (414.5 N) were all higher than that at week 0, and Fmax reached 630.0 N at week 20. This late rise reflected densification/compaction of the degraded, fragmented anchor during compression rather than an improvement in material strength; the load plateaued at approximately 610–630 N while displacement continued to increase. Unlike the progressive decline in molecular weight, compression Fmax remained high even at stages when appreciable chain scission had already occurred, indicating that the macroscopic mechanical response lagged behind molecular chain degradation.

Intrinsic viscosity decreased continuously with degradation time, consistent with the GPC molecular-weight decline and further supporting the occurrence of continuous chain scission. Representative load–displacement curves (Figure 2E) showed marked changes in shape at weeks 0, 8, 16, and 20, reflecting the evolution of anchor structural integrity during degradation. Photographs of the compression-testing setup are shown in Figure 2D.

### 3.3 Degradation-structure-mechanical correlations

We correlated Mn retention with compression Fmax retention (Figure 3A). The deviation of each time point from the 1:1 reference line (dashed) reflects the asynchrony between chain scission and mechanical deterioration. During early degradation (0–8 weeks), data points deviated clearly toward the Mn-retention side, indicating that chain scission preceded the decline in compression performance. As degradation proceeded, the gap between the two gradually narrowed.

Thermal analysis (Figure 3B) showed that Tg remained approximately 65 °C throughout degradation, with no melting endotherm, confirming that the material remained amorphous.

The structure-mechanical relationship is illustrated schematically in Figure 3C: in stage I (0–8 weeks), polymer chains remained largely intact and anchor mechanics were largely maintained; in stage II (8–16 weeks), random chain scission occurred and Mn declined markedly, yet the anchor could still bear load, reflecting structural changes that preceded the mechanical response; in stage III (16–24 weeks), molecular weight fell below a critical threshold and macroscopic mechanical deterioration occurred. The surface SEM time series (Figure 3D) provided microscopic morphological support: the surface was still relatively dense at week 4, micropores and cracks appeared at weeks 8–12, pores increased markedly at week 16, and extensive pitting was observed at week 24.

### 3.4 Acute-phase systemic and local response in animals

Samples were collected from SD rats at Day 8 after surgery. Body-weight changes showed no apparent intergroup separation (Figure 5A); the sham and suture groups remained generally stable or increased slightly in body weight after surgery.

Hematological measurements (Figure 5B) showed that all parameters in the sham and suture groups were within the normal reference ranges. In two anchor-group rats (anchor-1 and SD-06), individual parameters deviated from the normal reference ranges (see Figure 5B for the specific parameters). Serum biochemical measurements (Figure 5C) showed that ALT, AST, UREA, and CRE were generally within the normal reference ranges for SD rats across groups; the ALT level of anchor-1 was slightly higher than that of the other animals. The sham, suture, and anchor groups each contained n = 2 animals; all data are presented as individual data points without between-group statistical testing.

### 3.5 Local tissue response and histological evaluation

Wound-healing observations (Figure 6A) showed normal healing from Day 1 to Day 8 in the sham and suture groups, whereas partial wound dehiscence was observed in the anchor group at Days 3–5.

Gross observation of abdominal adhesion (Figure 6B) showed adhesion scores ≤1 (mild or no obvious adhesion) in the sham and suture groups. All three anchor-group rats had an adhesion score of 3, and anchor-2 showed severe adhesion with local encapsulation. Implant-region force-displacement results are shown in Figure 6C; the data for each group are presented as individual data points, with inter-animal variation in the anchor group.

Histological evaluation (Figure 7) showed normal skeletal muscle morphology in the sham and suture groups on HE staining and largely normal collagen distribution on Masson staining. In the anchor group, HE staining (40× fields) revealed skeletal muscle morphology in the implant region, and anchor-2 exhibited a local tissue defect consistently observed across HE and Masson staining. On Masson staining, focal collagen accumulation was observed in the suture group, and collagen distribution in the anchor group corresponded to the implant location. CD206 immunohistochemistry showed relatively sparse positive signals in all groups, with focal, scattered distribution and no diffuse staining or aggregation; a small number of suspected brownish signals were observed in anchor-2, and their specificity requires manual review.

Descriptive quantitative counting was performed in three non-overlapping fields per animal at ×40 magnification (FN = 22, field area 0.2376 mm^2^) (Figure 7D). HE-derived inflammatory cell density was 340.3–375.0 cells/mm^2^ in the sham group, 743.0–812.7 cells/mm^2^ in the suture group, and 775.3–882.0 cells/mm^2^ in the anchor group; the suture and anchor groups were generally higher than the sham group, and anchor-2 showed the highest density (882.0 cells/mm^2^). CD206-positive cell densities were low in all groups: 1.4–8.4 cells/mm^2^ in the sham group, 2.8–5.6 cells/mm^2^ in the suture group, and 1.4–5.6 cells/mm^2^ in the anchor group, all below 10 cells/mm^2^, with no clear trend related to implant type. These quantitative results are presented descriptively as individual animal values and ranges, without between-group statistical testing.

### 3.6 Mechanical enhancement by PCL coating

The PCL10% coating preserved the compression mechanical properties of the anchor through degradation-induced crystallization (Figure 1). The schematic illustration (Figure 1A) explains that amorphous PCL undergoes preferential hydrolysis during degradation, while crystalline regions are retained and form a physical crosslink network that compensates for the mechanical loss caused by the molecular-weight decline of the anchor matrix.

DSC analysis (Figure 1B) showed that ΔHm of the PCL10% coating was about 46 J/g initially (Xc ≈ 33%) and increased further after 4 weeks of degradation, indicating a marked increase in crystallinity. In contrast, the PCL5% coating showed only a weak crystallization signal after 4 weeks (ΔHm ≈ 0), suggesting that most of the thin coating had degraded; the PCL20% coating retained some crystallization signal, but it was weaker than that of the PCL10% group.

Compression Fmax retention showed that the PCL10% coating outperformed PCL5% and PCL20% (Figure 1C). GPC data at 12 weeks further showed that the Mn and Mw of the PCL10% group decreased to 5,713 Da and 9,804 Da, respectively, representing the lowest molecular weights among the three groups, yet its compression retention was the highest. This suggests that the physical crosslinks formed by crystallization compensated for molecular chain degradation. Suspected outliers in compression Fmax, specifically PCL5% at 12 weeks and PCL10% at 4 weeks, were retained in the figure and marked with open symbols. Mass-change data (Figure 1D) showed a gradual decrease in mass of the coated groups during degradation, consistent with the GPC results.

## 4 Discussion

This study systematically characterized the full degradation-structure-mechanical trajectory of uncoated PLGA anchors over 24 weeks *in vitro*, evaluated the regulatory effect of PCL coatings on mechanical performance under identical degradation conditions, and provided preliminary short-term *in vivo* safety and local tissue-response data in SD rats.

Structural degradation preceded the mechanical response along the degradation pathway. Uncoated anchors exhibited clear stage-wise degradation: during the first 8 weeks, an induction period, molecular weight was relatively well maintained (Mn retention >70%), mass loss was mild (<5.4%), and mechanical properties were largely preserved. From 8 to 16 weeks, degradation accelerated, with Mn falling sharply to 57.0%, while Tg remained stable at approximately 65 °C, indicating hydrolytic chain scission without crystallization. After 16 weeks, mass loss accelerated (>35%) and the structural integrity of the anchor was compromised. Throughout this process, however, compression Fmax did not follow the decline in molecular weight. When Mn had fallen to 57% of its initial value at week 12, Fmax was still not below its initial level, indicating that the macroscopic mechanical response clearly lagged behind molecular chain scission. This phenomenon has a well-established basis in degradable polyesters: under bulk degradation, random cleavage of ester bonds must accumulate to a critical molecular-weight threshold before macroscopic mechanical failure occurs. Our Mn-Fmax correlation analysis (Figure 3A) and the evolution of the load-displacement curves (Figure 2D) provide direct mechanical evidence for this “chain scission first, macroscopic mechanical change later” mechanism.

The amorphous, non-crystallizing nature of the material was further supported by consistent evidence from DSC and morphological observations. DSC showed a stable Tg of approximately 65 °C and no melting endotherm throughout degradation, confirming that the material remained amorphous. The surface SEM time series (Figure 3D) directly showed early surface micropore formation and later pore expansion, consistent with the kinetic trends in mass loss and molecular weight.

The PCL10% coating achieved mechanical compensation through degradation-induced crystallization. Among the three PCL coating concentrations, only PCL10% produced marked degradation-induced crystallization: ΔHm was about 46 J/g initially (Xc ≈ 33%) and increased after 4 weeks of degradation, indicating a substantial rise in crystallinity. The physical crosslink network formed by the retained crystalline regions effectively compensated for the mechanical loss caused by matrix molecular-weight decline. GPC data at 12 weeks showed that the PCL10% group had the lowest molecular weight among the three groups (Mn = 5,713 Da), yet it exhibited the highest compression retention, supporting the effectiveness of the physical-crosslink compensation mechanism. In contrast, the PCL5% coating showed only a very weak crystallization signal after 4 weeks (ΔHm ≈ 0), indicating that the thin coating had largely degraded early and could not form a stable crystalline network. The PCL20% coating retained some crystallization signal, but it was weaker than that of PCL10%, and both its molecular weight and mechanical performance had deteriorated markedly by 12 weeks. These results indicate an optimal window between PCL concentration and crystallization efficiency: a coating that is too thin cannot form a sufficient crystalline network, whereas one that is too thick may deviate from optimal performance because of differences in coating integrity and degradation kinetics.

The boundaries of the *in vivo* data must be stated explicitly. The animal experiments in this study were intended to provide short-term safety evidence rather than efficacy evaluation. Systemic safety analyses included n = 2 animals per group, and histological analyses included n = 2–3 animals per group; all data are presented as individual data points and cannot support between-group statistical testing. The anchor group differed from the sham and suture groups in wound healing, abdominal adhesion score, and some blood parameters, but given the small sample size, these differences only suggest a direction of local tissue response, are not statistically meaningful, and cannot be used to infer clinical safety. HE/Masson histological evaluation provided morphological descriptions of skeletal muscle morphology and collagen distribution in the implant region. Descriptive quantitative counting (Figure 7D) showed that HE-derived inflammatory cell density was lower in the sham group (∼340.3–375.0 cells/mm^2^) than in the suture (∼743.0–812.7 cells/mm^2^) and anchor (∼775.3–882.0 cells/mm^2^) groups, consistent with the direction of implant-related local inflammation. CD206-positive cell densities were low in all groups (sham 1.4–8.4, suture 2.8–5.6, and anchor 1.4–5.6 cells/mm^2^; all <10 cells/mm^2^) and showed no clear trend related to implant type. Because of the small sample size, manual interpretation, and field selection, these counts serve only as descriptive reference values and do not support between-group statistical inference; CD206 staining also cannot confirm M2 macrophage specificity.

Limitations. First, the compression mechanical time series and the samples used for mass loss, GPC, and animal testing originated from different production batches. Although all anchors were produced using the same PLGA formulation and manufacturing process with controlled dimensions, batch-to-batch material variation could not be fully excluded and may affect the continuity of the reported structure-mechanical correlations. Second, compression Fmax for the single PCL coatings was n = 1 at most time points, and suspected outliers were present for PCL5% at 12 weeks and PCL10% at 4 weeks; adding replicate specimens is necessary to validate the mechanical regulatory effect of the coatings in the future. Third, the GPC data for PCL-coated groups at 12 weeks were single-time-point data without coverage at 0/4/8 weeks, so the full-time-course trends in molecular weight for each coated group remain unclear. Fourth, the tensile test measured the implant region (fascia plus surrounding tissue) rather than the isolated fascia healing strength, and some anchors had partially migrated into the muscle layer by sampling; this method still needs to be standardized. Fifth, this study did not track the material properties of anchors after *in vivo* degradation (e.g., mass, Mn, or compression Fmax of retrieved anchors), so a quantitative in vitro-in vivo degradation bridge could not be established. Sixth, the histological cell-density counts are descriptive data based on manual interpretation with a small sample size and no between-group statistical testing; the robustness of the quantitative results needs to be verified with larger sample sizes and standardized interpretation criteria. Seventh, because the compression testing of single PCL coatings was n = 1 and the *in vivo* groups were n = 2–3, no inferential statistics were performed; the results are therefore descriptive and should be interpreted as preliminary.

In summary, the core contribution of this study is the identification of the asynchrony between molecular chain scission and macroscopic mechanical response during PLGA anchor degradation, together with the demonstration that a PCL10% coating can preserve mechanical performance through degradation-induced crystallization. The *in vivo* data provide preliminary morphological and descriptive quantitative reference information on short-term safety and local tissue response. Future work should perform replicate-specimen mechanical evaluation and full-time-course GPC tracking for PCL-coated groups using anchors from the same production batch, and should complement *in vivo* retrieval with *ex vivo* material-property testing after increasing the animal sample size, thereby providing a higher-confidence data chain for clinical translation.

## 5 Conclusion

This study systematically characterized the *in vitro* degradation pathway of uncoated PLGA suture anchors over 24 weeks and evaluated the mechanical regulation effect of PCL coatings under identical conditions, complemented by preliminary *in vivo* safety data in a rat fascia defect model.

The degradation of uncoated anchors followed a staged pattern in which molecular chain scission preceded macroscopic mechanical change. Mass loss remained modest during the first 8 weeks, accelerated after 16 weeks, while molecular weight declined progressively and the medium pH remained near neutral (approximately 7.2–7.4). Compression Fmax remained at or above its initial value over 0–16 weeks despite ongoing chain scission, indicating that mechanical deterioration lagged behind molecular degradation. This structural–mechanical decoupling was corroborated by the stable amorphous Tg (approximately 65 °C) and SEM observation of progressive surface pore formation.

Among the three PCL coating concentrations tested, PCL10% most effectively preserved compression performance through degradation-induced crystallization. The increase in ΔHm from about 46 J/g initially (Xc ≈ 33%) after 4 weeks of degradation demonstrated the formation of a crystalline physical crosslink network that compensated for molecular weight loss. In contrast, PCL5% degraded too rapidly to establish a stable crystalline network, and PCL20% showed no corresponding mechanical benefit.

*In vivo* evaluation at Day 8 post-surgery showed individual deviations in hematological and biochemical parameters and notable local tissue reactions, including wound dehiscence and grade 3 adhesions; these acute-phase findings cannot establish long-term systemic safety and require cautious interpretation given the small sample size (n = 2 per group). Histological evaluation combined qualitative morphology with descriptive quantitative counting: HE-derived inflammatory cell density was lower in the sham group (∼340–375 cells/mm^2^) than in the suture (∼743–813 cells/mm^2^) and anchor (∼775–882 cells/mm^2^) groups, whereas CD206-positive cell densities remained low in all groups (<10 cells/mm^2^), with sparse, unconfirmed signals in anchor specimens. No between-group inferential statistics were performed.

These findings establish the structure–mechanical relationship governing PLGA anchor degradation and support the feasibility of PCL coating-mediated mechanical preservation. They also define clear boundaries: the small animal sample size precludes statistical inference on safety margins, the histological cell-density data are descriptive and based on manual counting with a small sample, the compression time series originates from a different anchor production batch than the other degradation data, and the PCL coating compression dataset requires replication to substantiate the crystallization compensation mechanism.

## Data Availability

The raw data supporting the conclusions of this article will be made available by the authors, without undue reservation.
